# Simoctocog Alfa (Nuwiq) in Previously Untreated Patients with Severe Haemophilia A: Final Results of the NuProtect Study

**DOI:** 10.1055/s-0040-1722623

**Published:** 2021-02-13

**Authors:** Ri J. Liesner, Aby Abraham, Carmen Altisent, Mark J. Belletrutti, Manuel Carcao, Manuela Carvalho, Hervé Chambost, Anthony K. C. Chan, Leonid Dubey, Jonathan Ducore, Michael Gattens, Paolo Gresele, Yves Gruel, Benoit Guillet, Victor Jimenez-Yuste, Lidija Kitanovski, Anna Klukowska, Sunil Lohade, Maria Elisa Mancuso, Johannes Oldenburg, Anna Pavlova, Berardino Pollio, Marianne Sigaud, Vladimir Vdovin, Kateryna Vilchevska, John K. M. Wu, Martina Jansen, Larisa Belyanskaya, Olaf Walter, Sigurd Knaub, Ellis J. Neufeld

**Affiliations:** 1 Great Ormond Street Hospital for Children NHS Trust Haemophilia Centre, NIHR GOSH BRC, London, United Kingdom; 2 Department of Hematology, Christian Medical College, Vellore, India; 3 Unitat d'Hemofilia, Hospital Vall D'Hebron, Barcelona, Spain; 4 Pediatric Hematology, Department of Pediatrics, University of Alberta, Edmonton, Canada; 5 Division of Haematology/Oncology and Child Health Evaluative Sciences, Department of Paediatrics, Research Institute, Hospital for Sick Children, Toronto, Canada; 6 Congenital Coagulopathies Reference Centre, São João University Hospital Centre, Porto, Portugal; 7 AP-HM, Department of Pediatric Hematology Oncology, Children Hospital La Timone, Aix Marseille Univ, INSERM, INRA, C2VN, Marseille, France; 8 Division of Pediatric Hematology/Oncology, McMaster University, Hamilton, Canada; 9 Department of Pediatrics, Western Ukrainian Specialized Children's Medical Centre, Lviv, Ukraine; 10 Department of Pediatrics, University of California Davis Medical Center, Sacramento, United States; 11 Department of Paediatric Haematology and Oncology, Addenbrooke’s Hospital, Cambridge University Hospital NHS Foundation Trust, Cambridge, United Kingdom; 12 Department of Medicine and Surgery, University of Perugia, Perugia, Italy; 13 Centre Régional de Traitement de l'Hémophilie, Hôpital Trousseau, Tours, France; 14 Haemophilia Treatment Centre, Univ Rennes, CHU Rennes, Inserm, EHESP, Irset (Institut de recherche en santé, environnement et travail) - UMR_S 1085, Rennes, France; 15 Servicio de Hematología, Hospital Univeristario La Paz, Autónoma University, Madrid, Spain; 16 Department of Haemato-Oncology, University Medical Center Ljubljana, Ljubljana, Slovenia; 17 Department of Pediatrics, Haematology and Oncology, Warsaw Medical University, Warsaw, Poland; 18 Department of Hematology, Sahyadri Speciality Hospital, Pune, India; 19 Center for Thrombosis and Hemorrhagic Diseases, Humanitas Clinical and Research Center - IRCCS, Rozzano, Milan, Italy; 20 Institute of Experimental Haematology and Transfusion Medicine, University Clinic Bonn, Bonn, Germany; 21 Department of Transfusion Medicine, Regina Margherita Children Hospital of Turin, Turin, Italy; 22 Centre Régional de Traitement de I'Hémophilie, University Hospital of Nantes, Nantes, France; 23 Department of Hematology, Morozovskaya Children's Hospital, Moscow, Russian Federation; 24 Department of Hematology, State Institution “Institute of Urgent and Reconstructive Surgery named after V.K. Gusak of National Academy of Medical Sciences of Ukraine,” Donetsk, Ukraine; 25 British Columbia Children's Hospital, Vancouver, Canada; 26 Octapharma Pharmazeutika Produktionsges.mbH, Vienna, Austria; 27 Octapharma AG, Lachen, Switzerland; 28 St. Jude Children's Research Hospital, Memphis, Tennessee, United States

**Keywords:** coagulation, FVIII inhibitors, haemophilia

## Abstract

**Introduction** FVIII inhibitor development is the most serious contemporary treatment complication in haemophilia A, particularly in previously untreated patients (PUPs). No inhibitors developed in clinical trials in previously treated patients treated with simoctocog alfa (Nuwiq), a fourth-generation recombinant FVIII produced in a human cell line.

**Methods** The NuProtect study investigated the immunogenicity of simoctocog alfa in PUPs. NuProtect was a prospective, multinational, open-label, non-controlled, phase III study. PUPs with severe haemophilia A (FVIII:C <1%) of any age and ethnicity were treated with simoctocog alfa for 100 exposure days or a maximum of 5 years. Patients were true PUPs without prior exposure to FVIII concentrates or blood components. Inhibitor titres were measured with the Nijmegen-modified Bethesda assay; cut-off for positivity was 0.6 BU mL ^−1^ (≥0.6 to <5 low-titre, ≥5 high titre).

**Results** A total of 108 PUPs with a median age at first treatment of 12.0 months (interquartile range: 8.0–23.5) were treated with simoctocog alfa. *F8* mutation type was known for 102 patients (94.4%) of whom 90 (88.2%) had null *F8* mutations and 12 (11.8%) had non-null mutations. Of 105 PUPs evaluable for inhibitor development, 28 (26.7%) developed inhibitors; 17 high titre (16.2%) and 11 low titre (10.5%). No PUPs with non-null *F8* mutations developed inhibitors.

**Conclusion** In the NuProtect study, the rate of inhibitor development in PUPs with severe haemophilia A treated with simoctocog alfa was lower than the rate reported for hamster-cell-derived recombinant factor VIII products in other recent clinical trials. No inhibitors were reported in PUPs with non-null *F8* mutations.

## Introduction

Haemophilia A is an X-linked bleeding disorder that is usually diagnosed in infancy, especially in severe cases (factor VIII coagulant activity [FVIII:C] <1% of normal). 1 Patients with haemophilia A require life-long treatment to prevent or control bleeding. 2 3

When FVIII treatment is started, previously untreated patients (PUPs) with severe haemophilia A face a period of high risk for development of FVIII-neutralising antibodies (inhibitors), which interfere with the haemostatic function of FVIII treatment. The highest risk is present usually during the first 20 exposure days (EDs) to exogenous FVIII but a residual risk persists until the completion of 75 EDs. 4 5 Inhibitors typically develop in up to 40% of PUPs 6 and are widely considered the most serious treatment-related complication of haemophilia A due to the detrimental impact on bleeding rates, mortality, quality of life and treatment costs. 7 8 9 10 Remaining free from inhibitors is important to enable effective FVIII treatment for bleeding events or surgery, as well as to remain eligible for potential future treatment options such as gene therapy. 6 11

Several patient- and treatment-related risk factors for inhibitor development have been identified, such as severity of haemophilia A, a family history of inhibitors, ethnicity, type of *F8* mutation, polymorphisms of immune response genes, treatment intensity and product type. 4 12 13 14 Whereas some reports suggest an increased inhibitor risk associated with the use of recombinant (r) FVIII compared with plasma-derived (pd) FVIII concentrates, 15 16 17 other reports suggest no difference in inhibitor risk with respect to product type. 18 19 20 The SIPPET study was the only prospective, randomised, controlled study to compare the immunogenicity of pdFVIII versus rFVIII (all derived from hamster cell lines). 21 The cumulative incidence of all and high-titre inhibitors in patients treated with pdFVIII was 26.8% (95% confidence interval [CI]: 18.4–35.2%) and 18.6% (95% CI: 11.2–26.0%), respectively. In patients treated with rFVIII, the cumulative incidence of all and high-titre inhibitors was 44.5% (95% CI: 34.7–54.3%) and 28.4% (95% CI: 19.6–37.2%), respectively. 21 As SIPPET enrolled patients between 2010 and 2014, rFVIII products licensed after 2014 were not included in the study.

Simoctocog alfa (Nuwiq; Octapharma AG) is a fourth-generation rFVIII product 22 manufactured using a human cell line without chemical modification or protein fusion, with the aim of reducing inhibitor development by replicating the native human FVIII protein and avoiding incorporation of potentially immunogenic elements of animal cell origin. 23 24 25 26 It contains only human post-translational modifications 24 and is free of any added human or animal impurities. 23 26 Simoctocog alfa is fully sulphated at Tyr1680, which confers a high binding affinity for its natural carrier protein, von Willebrand factor (VWF). 24 25 These properties of simoctocog alfa may lower its immunogenic potential by shielding potentially antigenic epitopes from recognition by the immune system and inhibiting uptake of FVIII by antigen-presenting cells. 12 Additionally, simoctocog alfa contains only human glycans and is devoid of potentially immunogenic glycan epitopes present in rFVIII products derived from hamster cell lines. 27 28 A combination of high VWF affinity and absence of antigenic glycans has the potential to reduce the overall immunogenic challenge. The efficacy and safety of simoctocog alfa were demonstrated in studies of previously treated patients (PTPs) with severe haemophilia A, and no inhibitors were reported in 201 PTPs, including 59 PTPs below 12 years of age. 29

The NuProtect study was initiated in 2013 to assess the immunogenicity of simoctocog alfa in patients without any previous exposure to FVIII concentrates or any blood products containing FVIII (true PUPs) who were treated with simoctocog alfa for 100 EDs or up to 5 years, whichever came first. Here we report the final inhibitor data of the NuProtect study; efficacy and safety data will be reported separately.

## Methods

Details of the study design and clinical assessments have been published previously 30 and are provided in brief here with respect to inhibitor development.

## Study Design and Patients

The NuProtect study ( www.clinicaltrials.gov NCT01712438; EudraCT 2012–002554–23) was a prospective, multicentre, multinational, open-label, non-controlled, phase III study. Male PUPs with severe haemophilia A (FVIII:C < 1%) of any age and ethnicity were enrolled and treated with simoctocog alfa from the first ED. Exclusion criteria included previous exposure to any FVIII concentrates or blood components, diagnosis of any coagulation disorder other than haemophilia A, concomitant treatment with systemic immunosuppressive drugs, participation in other interventional clinical studies currently or within previous 4 weeks and severe liver or kidney disease. The trial was approved by all relevant independent ethics committees and institutional review boards and was conducted in accordance with the ethical principles of the Declaration of Helsinki. Written informed consent was provided by the parent/legal guardian of all participants.

## Study Treatment

Patients received simoctocog alfa for prophylaxis or on-demand treatment, as well as for the treatment of breakthrough bleeding episodes during prophylaxis and to cover surgical procedures. The type of treatment and the dose were determined by investigators based on the clinical situation of the patient.

The recommended dose for prophylaxis was 20 to 50 IU FVIII kg ^−1^ and patients could switch between on-demand and prophylactic treatment during the study. Use of FVIII concentrates other than simoctocog alfa was prohibited, except in emergency situations. Patients permanently switching to another FVIII product within the study participation period were assessed as treatment failures in the efficacy analyses. The treatment period was 100 EDs or 5 years, whichever occurred first.

## Inhibitor Analysis

The primary endpoint of the study was the incidence of anti-FVIII inhibitors after simoctocog alfa administration. Inhibitor titres were measured in plasma by the Nijmegen-modified Bethesda assay (cut-off: 0.6 Bethesda units [BU] mL ^−1^ ). 31

Inhibitor activity was determined at a central laboratory (LabCorp Clinical Trials, Colorado, United States) at baseline (screening), every 3 to 4 EDs until ED 20, every 10 to 12 EDs or every 3 months ± 2 weeks after ED 20 and at study completion. Additionally, inhibitor activity was tested at any time during the study if inhibitor development was suspected. In the case of a positive inhibitor result, a retest of the first sample was done and a second blood sample was drawn and tested at the central laboratory. Inhibitors were confirmed if the retest or second sample was positive or a subsequent test at any time in the study was positive. The day of the first positive inhibitor test was used to calculate the time to inhibitor development. The definitions for inhibitor thresholds were ≥0.6 to <5 BU mL ^−1^ for low-titre and ≥5 BU mL ^−1^ for high-titre inhibitors. Inhibitors were regarded as transient if they occurred without any clinical symptoms, required no increase in FVIII dosing and decreased to <0.6 BU mL ^−1^ within a period of 6 months after first detection. Patients who developed a non-transient, low- or high-titre inhibitor were offered the option to start immune tolerance induction (ITI) treatment with simoctocog alfa within the study; ITI data are not reported here.

The incidence of FVIII inhibitors was examined according to patient-related variables: race, family history of inhibitors and *F8* mutation type (performed by J. Oldenburg and A. Pavlova, University Clinic Bonn, Bonn, Germany). Intron 22 inversions, intron 1 inversions, nonsense mutations, splice site mutations, small duplications, small deletions (excluding in-frame and within a poly-A run) and large deletions were classified as null mutations. All other mutations were classified as non-null mutations. The effect of treatment-related variables, including treatment regimen (prophylaxis vs. on-demand), surgery, peak treatment moments (≥3 subsequent days with FVIII dosing and/or at least 1 day with prophylactic doses of >50 IU kg ^−1^ ) and dose (<40 vs. ≥40 IU kg ^−1^ day ^−1^ ), were also examined.

## Statistical Analyses

No inferential analysis involving formal testing was planned in this non-controlled study. Therefore, no formal sample size estimation was performed, but the sample size was chosen to satisfy European Medicines Agency guidelines current at the time the study was initiated (EMA/CHMP/BPWP/144533/2009).

Statistical analyses were exploratory and were performed by Clinipace (Marburg, Germany). Absolute and cumulative inhibitor incidences were calculated. Cumulative incidences and 95% CIs were calculated using Kaplan–Meier methods relative to the population size and accounted for changes over time in the number of patients remaining at risk of developing inhibitors.

## Results

### Patients

**Fig. 1:**
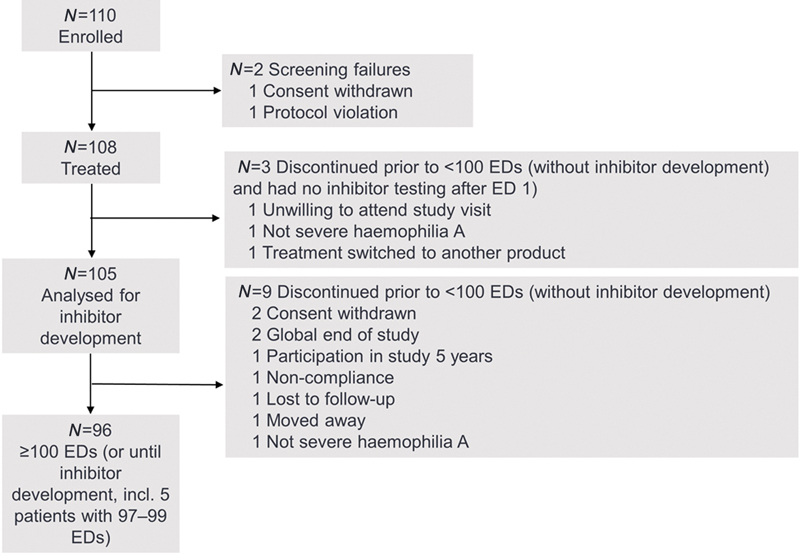
Patient disposition.

The study was conducted at 38 sites in 17 countries. A total of 110 patients were screened, 108 (98%) were treated with simoctocog alfa and 105 (97%) underwent inhibitor testing at least once during the study ( Fig. 1 ). Three patients discontinued the study after 1 ED without an inhibitor test and were not included in the inhibitor analysis. A total of 105 patients underwent inhibitor testing and were included in the immunogenicity analyses. The 105 patients were treated for a median of 100 EDs (interquartile range [IQR]: 15–102), excluding treatment for ITI. Ninety-six patients completed the study, i.e. received simoctocog alfa for ≥100 EDs without developing an inhibitor (including 5 patients with 97–99 EDs due to miscounting) and/or developed an inhibitor. Five patients who developed a transient inhibitor subsequently reached ≥100 EDs. Nine patients discontinued the study after a median of 14 EDs (IQR: 5–32) without developing an inhibitor.

Baseline demographics are shown in Table 1 . At ED 1, the median age of enrolled patients at first treatment was 12.0 months (IQR: 8.0–23.5), and 82 (75.9%) were aged ≤24 months. *F8* mutation type was known for 102/108 (94.4%) and 90 of them (88.2%) had null *F8* mutations. Patients with null mutations received their first treatment at a median age of 12.0 months (IQR: 8.0–22.8) and those with non-null mutations at a median of 14.0 months (IQR: 10.5–21.3). A family history of haemophilia was present in 42 (38.9%) patients of whom 13 (31.0%) also had a family history of inhibitors.

**Table 1:** Patient demographics

Parameter	All patients ( *N* = 108)
Age at first treatment, mo, median (IQR)	12.0 (8.0–23.5)
Age at first treatment, *N* (%)	
< 1 mo	1 (0.9)
1–6 mo	9 (8.3)
> 6–12 mo	47 (43.5)
> 12–24 mo	25 (23.1)
> 24 mo	26 (24.1)
Race, *N* (%)	
White	89 (82.4)
Asian	14 (13.0)
Native American/Alaska native	1 (0.9)
Other	4 (3.7)
Gene mutation defect, *N* (%)	
Intron 1 inversion	3 (2.8)
Intron 22 inversion	47 (43.5)
Large deletion	5 (4.6)
Missense	12 (11.1)
Nonsense	11 (10.2)
Small deletion	14 (13.0)
Small duplication	6 (5.6)
Splice site mutation	4 (3.7)
No mutation found	2 (1.9)
Missing	4 (3.7)
*F8* mutation type, a *N* (%)	
Null mutations	90 (88.2)
Non-null mutations b	12 (11.8)
Family history of haemophilia, *N* (%)	42 (38.9)
Family history of inhibitors, *N* (%)	13 (31.0 c )

Abbreviation: IQR, interquartile range.

a Data for 102 patients with genotype classification.

b All non-null mutations were missense mutations.

c Percentage of those with a family history of haemophilia.

### Inhibitor Development

Of the 105 patients who underwent inhibitor testing, 28 (26.7%) developed inhibitors, which were high titre in 17 (16.2%) and low titre in 11 (10.5%). Five of the 11 patients with low-titre inhibitors had transient inhibitors with titres becoming undetectable without the need for treatment regimen modification and all were subsequently treated for ≥100 EDs. In 97 patients who were treated for ≥75 EDs (or developed an inhibitor), the absolute inhibitor incidences were 28.9, 17.5 and 11.3%, for all, high- and low-titre inhibitors, respectively.

**Fig. 2:**
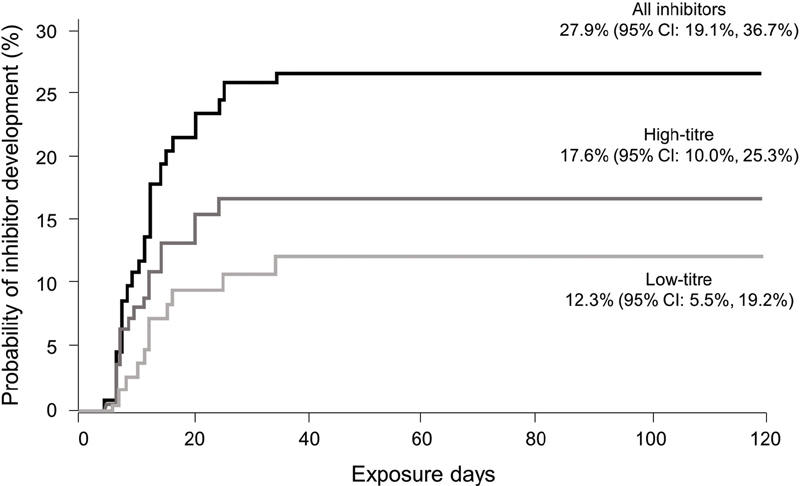
Cumulative incidence of inhibitor development. Kaplan–Meier estimate.

The cumulative inhibitor incidence was 27.9% (95% CI: 19.1–36.7%) for all inhibitors, 17.6% (95% CI: 10.0–25.3%) for high-titre inhibitors and 12.3% (95% CI: 5.5–19.2%) for low-titre inhibitors ( Fig. 2 ).

The median (IQR) peak (maximum) inhibitor titre was 154.8 (38.2–300.5) BU mL ^−1^ during the study for high-titre inhibitors and 3.0 (2.3–4.0) BU mL ^−1^ for low-titre inhibitors. The median (range) time to inhibitor development was 11.0 (4–34) EDs for all inhibitors (9.0 [4–24] EDs and 12.0 [6–34] EDs for high- and low-titre inhibitors, respectively). Inhibitors developed after ED 20 in only 3 cases (1 high titre [ED 24], 2 low titre [ED 25 and ED 34]).

**Fig. 3:**
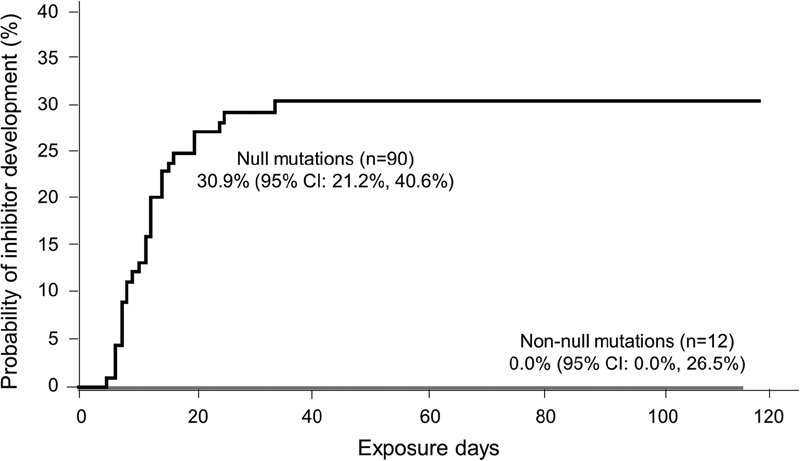
Cumulative incidence of inhibitor development by null or non-null *F8* gene mutation. Kaplan–Meier estimate.

**Fig. 4:**
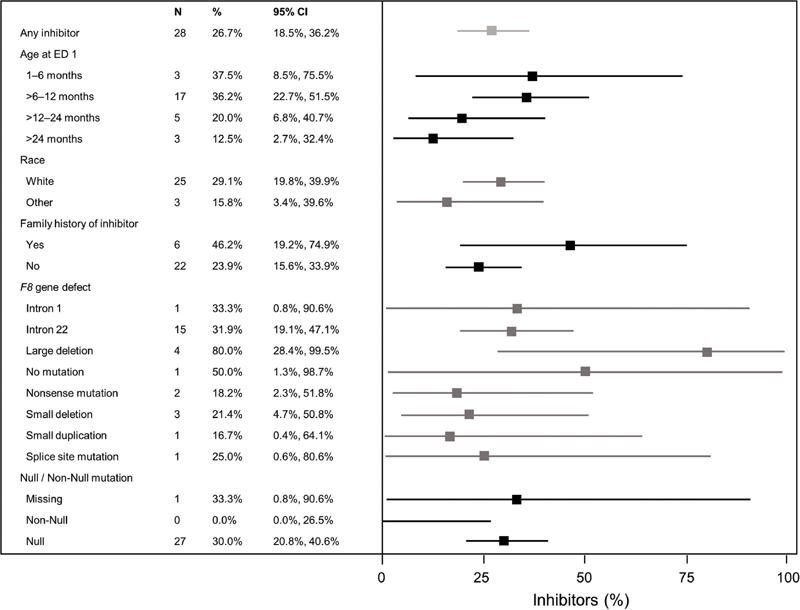
Incidence of inhibitors in subgroups. Forest plot of absolute incidence percentages including exact 95% confidence interval.

Of 90 patients with null *F8* mutations, 27 (30.0%) developed inhibitors of which 17 (18.9%) were high titre (no mutation was found for one patient with a low-titre inhibitor); the cumulative incidence was 30.9% (95% CI: 21.2–40.6%) for all inhibitors and 20.3% (95% CI: 11.6–28.9%) for high-titre inhibitors ( Fig. 3 ). No patients with non-null *F8* mutations ( *n* = 12) developed inhibitors. Patients with large *F8* deletions ( *n* = 5) had the highest inhibitor incidence (80%) ( Fig. 4 ). Patients with a family history of inhibitors ( *n* = 13) had a higher incidence of inhibitors (46.2%) compared with those without ( *n* = 92) (23.9%; Fig. 4 ). Patients aged 1 to 6 months (37.5%; *n* = 8) and aged >6–12 months (36.2%; *n* = 47) at first treatment had a higher incidence of inhibitors compared with patients aged >12–24 months (20.0%; *n* = 25) or >24 months (12.5%; *n* = 24) ( Fig. 4 ).

Treatment-related factors (treatment regimen, surgery, peak treatment moments and dose) did not have a significant effect on the incidence of inhibitors up to ED 35 or over the whole study period (data not shown).

## Discussion

We present the final results on inhibitor development in the NuProtect study with simoctocog alfa, a fourth-generation human cell-line-derived rFVIII. This analysis reports inhibitor data for 105 PUPs treated with simoctocog alfa, making it the largest prospective study in true PUPs with a single FVIII product. The absolute incidence of all and high-titre inhibitors in NuProtect was 26.7 and 16.2%, respectively. The cumulative incidence of all and high-titre inhibitors was 27.9 and 17.6%, respectively. These rates are considerably lower than the rates reported for the rFVIII arm in the SIPPET study, all derived from hamster cell lines (cumulative incidence 44.5% for all inhibitors and 28.4% for high-titre inhibitors), and in line with results obtained for the pdFVIII arm of the SIPPET study (26.8 and 18.6% for all and high-titre inhibitors, respectively). 21

Recently published phase III PUP trials 32 33 addressing the risk of inhibitor development with other single rFVIII products (all derived from hamster cell lines) have reported inhibitor rates in line with those reported for the rFVIII arm of the SIPPET study and hence higher than those reported with simoctocog alfa in our study. In a study of 58 PUPs treated with turoctocog alfa (NovoEight), 25 (43.1%) developed inhibitors of which 16 (27.6%) were high titre. 32 In a study of 23 PUPs treated with single-chain rFVIII (Afstyla), 12 (52%) developed inhibitors of which 6 (26%) were high titre. 33

**Fig. 5:**
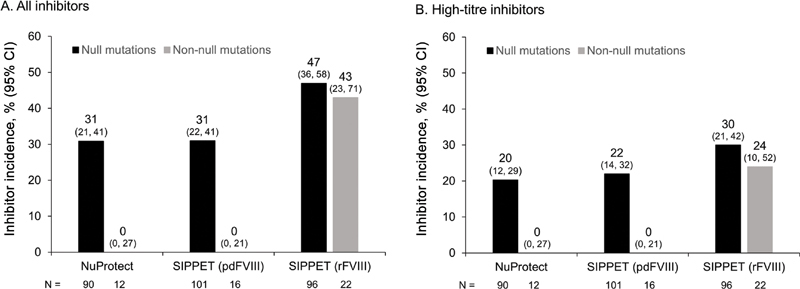
Cumulative inhibitor incidence in the current study (NuProtect) and the SIPPET 34 study. Data are shown by *F8* mutation (null or non-null) for all inhibitors ( **A** ) and high-titre inhibitors ( **B** ).

**Fig. 6:**
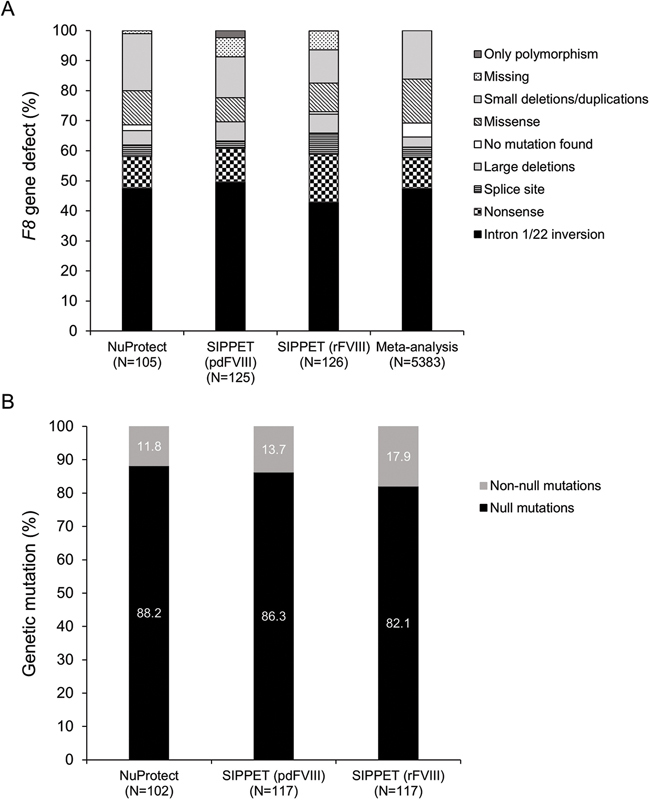
Distribution of genetic defects. ( **A** ) *F8* gene defect in the current study (NuProtect), the SIPPET study, 21 and a meta-analysis 13 of patients with severe haemophilia A and ( **B** ) percentage null or non-null mutations in NuProtect and SIPPET. 21

*F8* mutation type had an influence on the risk of inhibitor development in our study as has been previously reported. 13 None of the 12 patients with non-null *F8* mutations developed inhibitors with simoctocog alfa, which is consistent with what was reported for patients treated with pdFVIII in a post-hoc analysis of SIPPET data. 34 Among SIPPET patients with null *F8* mutations, the cumulative incidence of inhibitors was 31% in 101 patients treated with pdFVIII and 47% in 96 patients treated with rFVIII. Among SIPPET patients with non-null *F8* mutations, no inhibitors developed in 16 patients receiving pdFVIII treatment, whereas the cumulative incidence of inhibitors was 43% in 22 patients treated with rFVIII. 34 These results suggest that simoctocog alfa appears to follow the pattern exhibited by pdFVIII concentrates rather than that of the hamster-cell derived rFVIII concentrates ( Fig. 5 ). Notably, the distribution of *F8* mutations was similar in NuProtect and SIPPET ( Fig. 6 ).

There is evidence in the literature that a family history of inhibitors is associated with an increased risk of inhibitor development. 35 In NuProtect, the incidence of inhibitors in patients with a family history of inhibitors (46.2%; *n* = 13) was double that of patients without a family history (23.9%; *n* = 92). This difference was not statistically significant, but this may have been influenced by the relatively low number of patients with a family history of inhibitors.

In NuProtect, age ≤12 months at first treatment was an independent prognostic factor for the development of inhibitors. PUPs aged 1 to 6 months at first treatment had the highest risk and those aged >24 months had the lowest risk for developing inhibitors. There are conflicting published data regarding the impact of age at first treatment on the risk for inhibitor formation. In two cohort studies, the risk of inhibitor development was higher in younger patients, but these studies were not controlled for other risk factors. 36 37 Later studies that adjusted for confounders did not report an effect of age on inhibitor risk. 16 38

There is also some evidence in the literature to suggest that treatment-related factors, particularly intensity of treatment, influence inhibitor development. 14 39 In our study, treatment-related factors did not have a significant effect on inhibitor risk, which we speculate might be indicative of a lower immunogenic potential of Nuwiq.

A limitation of the NuProtect study is that almost all patients (95.4%) enrolled across 17 countries were white or Asian, and no patients of African origin who have been reported to be at higher risk of inhibitor development 40 41 were enrolled. A strength of the study is the inclusion of only true PUPs, i.e., without prior exposure to FVIII concentrates or any other blood products that may otherwise have mitigated or confounded the risk attributable to the study product. In addition, extensive immunogenicity testing was done throughout the study and all analyses were performed at a central laboratory.

In conclusion, the data from the NuProtect study show that PUPs with severe haemophilia A treated with simoctocog alfa had a lower high-titre inhibitor rate than that reported for hamster-cell-derived rFVIII products in other recent clinical trials and no inhibitors were observed in patients with non-null *F8* gene mutations.

## Notes

**What is known about this topic?**

- FVIII inhibitor development in previously untreated patients (PUPs) is the most serious contemporary treatment complication in haemophilia A.
- FVIII inhibitors develop in up to 40% of PUPs and have a detrimental impact on bleeding rates, quality of life and treatment costs.

**What does this paper add?**

- The NuProtect study was a multinational, prospective, open-label, phase III study investigating the immunogenicity of simoctocog alfa (Nuwiq), a recombinant FVIII produced in a human cell line, in PUPs.
- Inhibitors developed in 26.7% of 105 PUPs treated with simoctocog alfa; high-titre inhibitors in 16.2%.
- The NuProtect study is the largest prospective study in PUPs with a single FVIII product.
- The study demonstrates that PUPs with severe haemophilia A treated with simoctocog alfa had a lower rate of high-titre inhibitor development than that reported for rFVIII products in other recent clinical trials.
